# PSYCHOSOCIAL STATUS, RESILIENCE, AND DEPRESSION OF OLDER CAREGIVERS IN TAIWAN

**DOI:** 10.1093/geroni/igz038.1888

**Published:** 2019-11-08

**Authors:** Tsuann Kuo, Hei-Yin Ng, Der-Yan Han

**Affiliations:** 1 Chung Shan Medical University, Taichung City, Taiwan; 2 Taipei Medical University, Taipei, Taiwan, Taiwan; 3 Taipei Medical University, Taipei City, Taiwan

## Abstract

A rapidly increasing older population and changing family structures in Taiwan have resulted in a significant shift in caregiving resources; there are now more older caregivers, and their psychosocial well-being and quality of life may be affected as a result of their new responsibilities. This study used secondary data from the 2018 Survey of Elderly Mental Health (N=1,113) to examine the psychosocial status, resilience, and depression of caregivers who were 65 years old and older. The results showed that the typical older caregiver was 74.15 years old, likely to be married and living with a spouse (63.6%), retired (57.4%), and had a monthly income less than 30,000 NT (30.7%). Compared with caregivers under 65 years of age, older caregivers had higher co-morbidity and lower quality of life as well as greater resilience and higher levels of depression. Both positive emotions (p<0.05) and self-assessed health (p<0.001) were found to be significant variables affecting depression and resilience. And negative emotions (p<0.001) also influenced depression. This study was the first in the nation to tackle issues related to aging caregivers that can identify policy, research, and practice implications to improve the general health and lives of older caregivers. Future research can use the results to explore opportunities for older caregivers that will minimize negative emotions in order to build resilience and combat depression.

